# The Evolutionarily Conserved Mediator Subunit MDT-15/MED15 Links Protective Innate Immune Responses and Xenobiotic Detoxification

**DOI:** 10.1371/journal.ppat.1004143

**Published:** 2014-05-29

**Authors:** Read Pukkila-Worley, Rhonda L. Feinbaum, Deborah L. McEwan, Annie L. Conery, Frederick M. Ausubel

**Affiliations:** 1 Division of Infectious Diseases; Massachusetts General Hospital; Harvard Medical School; Boston, Massachusetts, United States of America; 2 Department of Molecular Biology; Massachusetts General Hospital; Harvard Medical School; Boston, Massachusetts, United States of America; 3 Department of Genetics; Harvard Medical School; Boston, Massachusetts, United States of America; University of Birmingham, United Kingdom

## Abstract

Metazoans protect themselves from environmental toxins and virulent pathogens through detoxification and immune responses. We previously identified a small molecule xenobiotic toxin that extends survival of *Caenorhabditis elegans* infected with human bacterial pathogens by activating the conserved p38 MAP kinase PMK-1 host defense pathway. Here we investigate the cellular mechanisms that couple activation of a detoxification response to innate immunity. From an RNAi screen of 1,420 genes expressed in the *C. elegans* intestine, we identified the conserved Mediator subunit MDT-15/MED15 and 28 other gene inactivations that abrogate the induction of PMK-1-dependent immune effectors by this small molecule. We demonstrate that MDT-15/MED15 is required for the xenobiotic-induced expression of p38 MAP kinase PMK-1-dependent immune genes and protection from *Pseudomonas aeruginosa* infection. We also show that MDT-15 controls the induction of detoxification genes and functions to protect the host from bacteria-derived phenazine toxins. These data define a central role for MDT-15/MED15 in the coordination of xenobiotic detoxification and innate immune responses.

## Author Summary

Metazoans respond to environmental threats in part through conserved pathways that coordinate protective transcriptional responses. During infection with an invasive pathogen, for example, innate immune pathways regulate the secretion of antimicrobial immune effectors. Likewise, exposure to toxic molecules leads to the induction of detoxification mechanisms that protect the host from the deleterious effects of these compounds. Here we find that a conserved transcriptional regulator MDT-15/MED15 links xenobiotic detoxification and immune responses in a manner that is important for protection during bacterial infection. We also show that MDT-15/MED15 is necessary for the host to resist the lethal effects of secreted toxins produced by pathogenic bacteria. Rapid coordination of these protective host responses through MDT-15/MED15 may therefore be part of a conserved survival strategy in the wild.

## Introduction

In nature, organisms encounter environmental insults, such as chemical toxins, secreted microbial virulence factors and invasive pathogens, that threaten their ability to survive and reproduce. As a result, metazoans have evolved protective pathways to counter these challenges. For example, gene families such as cytochrome P450s (CYPs), glutathione-s-transferases (GSTs), and UDP-glucuronosyltransferases (UDPs) detoxify xenobiotic small molecule toxins and are conserved from nematodes to humans [1]. Likewise, innate immune defenses provide protection from invasive pathogens [2]. Recent publications have suggested that recognition of xenobiotic toxins is involved in the activation of immune response pathways [3], [4]. From an evolutionary perspective, it is logical that hosts respond to threats encountered in the wild at least in part through surveillance pathways that monitor the integrity of core cellular machinery, which are often the targets of xenobiotic small molecules or microbe-generated toxins. These studies predict that organisms may integrate detoxification and immune responses as a means to respond rapidly to such challenges, but the mechanisms underlying this coordinated host response have not been reported.

Our research group and others use bacterial and fungal pathogenesis assays in the nematode *Caenorhabditis elegans* to investigate mechanisms of immune pathway activation in intestinal epithelial cells [2]. Genetic analyses of *C. elegans* that are hypersusceptible to bacterial infection have revealed that the nematode mounts defense responses through evolutionarily conserved innate immune pathways. For example, the *C. elegans* NSY-1/SEK-1/PMK-1 Mitogen Activated Protein (MAP) kinase pathway, orthologous to the ASK1 (MAP kinase kinase kinase)/MKK3/6 (MAP kinase kinase)/p38 (MAP kinase) pathway in mammals, is required for protection against pathogens [5]. *C. elegans* animals carrying loss-of-function mutations in this pathway have defects in the basal and pathogen-induced expression of immune effectors and are hypersusceptible to killing by bacterial and fungal pathogens [5]–[7].

We previously used a *C. elegans* pathogenesis assay as a means to identify small molecules that protect the host during bacterial infection [8]. One of the compounds identified in this screen, a small molecule called RPW-24, extended the survival of nematodes infected with the human bacterial pathogen *Pseudomonas aeruginosa* by stimulating the host immune response via the p38 MAP kinase PMK-1 pathway [9]. A genome-wide microarray analysis of animals exposed to RPW-24 revealed that, in addition to inducing the transcription of putative immune effectors, this molecule also strongly upregulated Phase I and Phase II detoxification enzymes (CYPs, GSTs and UDPs), suggesting that RPW-24 is a xenobiotic toxin to *C. elegans*. Consistent with this hypothesis, RPW-24 caused a dose dependent reduction of nematode lifespan on nonpathogenic food and delayed development of animals that were exposed starting at the first larval stage.

Here we sought to use RPW-24 as a tool to characterize mechanisms of p38 MAP kinase PMK-1 pathway activation in *C. elegans*. We found that activation of PMK-1-regulated pathogen response genes is genetically linked to the induction of genes involved in the detoxification of small molecule toxins. We show that the evolutionarily conserved Mediator subunit MDT-15/MED15 is required for the induction of the p38 MAP kinase PMK-1-mediated immune effectors as well as non-PMK-1-dependent detoxification genes by RPW-24. These data demonstrate that the host response to a xenobiotic involves coordination of detoxification and innate immune responses via the Mediator subunit MDT-15. Moreover, loss of MDT-15 function has important physiological effects on the ability of an animal to mount protective immune responses, resist bacterial infection and survive challenge from lethal bacterial toxins.

## Results

### RNAi Screen Identifies Regulators of p38 MAP Kinase PMK-1-Dependent Genes

To investigate mechanisms of immune activation in *C. elegans*, we generated a GFP transcriptional reporter for the immune response gene *F08G5.6*. *F08G5.6* is a putative immune effector that contains a CUB-like domain [6] and is transcriptionally induced by exposure of *C. elegans* to several bacterial pathogens, including *P. aeruginosa* [6], [10]. We chose *F08G5.6* for these studies because it is upregulated more than 100-fold by RPW-24 in a manner that requires the p38 MAP kinase PMK-1 [9].

**Figure 1:**
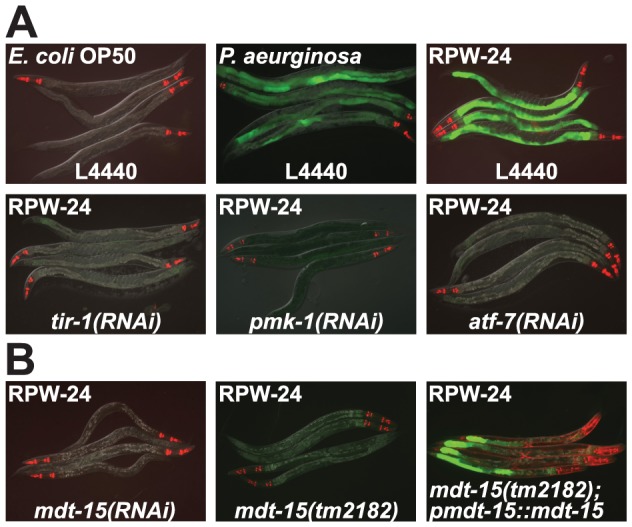
RNAi screen identifies a role for the *C. elegans* Mediator subunit MDT-15 in regulating the induction of the p38 MAP kinase PMK-1-dependent immune reporter *pF08G5.6::GFP*. (A) *C. elegans* carrying the *pF08G5.6::GFP* immune reporter were exposed to the vector control (L4440) or the indicated RNAi strain, and then transferred at the L4 stage to either *E. coli* OP50 food, *P. aeruginosa* or *E. coli* OP50 supplemented with RPW-24 for 18 hours. Photographs were acquired using the same imaging conditions. (B) *C. elegans mdt-15(RNAi)* and *mdt-15(tm2182)* animals carrying the *pF08G5.6::GFP* immune reporter with or without *agEx114* (*pmdt-15::mdt-15*) were exposed to RPW-24 as described above. In all animals shown in this figure, red pharyngeal expression is the *pmyo-2::mCherry* co-injection marker, which confirms the presence of the *pF08G5.6::GFP* transgene. In the *mdt-15(tm2182)* animals carrying the *agEx114* array, the presence of the *pmdt-15::mdt-15* transgene is confirmed by the *pmyo-3::mCherry* co-injection marker which is expressed in the body wall muscle.

*pF08G5.6::GFP* was induced in the *C. elegans* intestine during *P. aeruginosa* infection and GFP expression was also robustly upregulated in *pF08G5.6::GFP* animals following exposure to RPW-24 when animals were feeding on nonpathogenic *E. coli* (Figure 1A). When three components of the p38 MAP kinase PMK-1 signaling cassette were individually knocked down by RNAi [*tir-1* [11], *pmk-1* [6] and *atf-7* [12]], *pF08G5.6::GFP* induction by RPW-24 was entirely abrogated (Figure 1A).

The level of *F08G5.6* induction during bacterial infection is dependent upon the virulence of the invading pathogen (Figure S1). We exposed *C. elegans* to several *P. aeruginosa* strains, each of which was previously shown to have a different pathogenic potential toward nematodes [13] and used qRT-PCR to determine the expression levels of *F08G5.6* in these animals. In general, more pathogenic *P. aeruginosa* strains caused significantly greater induction of *F08G5.6*, suggesting that some aspect of *P. aeruginosa* virulence, rather than a structural feature of the bacteria itself, causes the activation of *F08G5.6*. Consistent with these data, it was previously shown that *C. elegans* primarily responds to virulence-related cues to mount its innate immune defenses towards *P. aeruginosa* [14]–[16].

To identify genes that regulate the p38 MAP kinase PMK-1 pathway in response to RPW-24, we screened a library of RNAi clones corresponding to 1,420 genes expressed in *C. elegans* intestinal epithelium (approximately 9% of the genome, Table S1B) for gene inactivations that abrogated the RPW-24-mediated induction of *pF08G5.6::GFP*. We specifically focused on intestinally expressed genes because of the recognized role for intestinal cells in coordinating the host response to ingested pathogens [2], [17], and because *P. aeruginosa* and RPW-24 induce *F08G5.6* expression in the intestine (Figure 1A). Our initial screening effort identified 153 genes that, when inactivated by RNAi, diminished or abrogated the induction of *pF08G5.6::GFP* by RPW-24.

We took several steps to identify specific regulators of p38 MAP kinase PMK-1-dependent immune effectors among these 153 gene inactivations. First, we noticed that knockdown of many of these genes markedly slowed nematode growth. To eliminate genes that simply reduced GFP reporter expression as a consequence of pleiotropic effects on worm growth and development, we determined if these 153 gene inactivations also affected induction of the *C. elegans* immune reporter *irg-1::GFP* [14]. *irg-1* is strongly upregulated in intestinal epithelial cells during *P. aeruginosa* infection or by an *E. coli* strain that expresses the bacterial virulence factor Exotoxin A (ToxA), but via a pathway independent of p38 MAP kinase PMK-1 signaling [14]–[16]. Moreover, RPW-24 does not cause the induction of *irg-1::GFP* and mutation of the *zip-2* gene, which encodes the transcription factor that regulates *irg-1* expression, does not affect the RPW-24-mediated induction of *F08G5.6* (data not shown) or alter the ability of RPW-24 to extend the survival time of nematodes infected with *P. aeruginosa* [9]. We therefore discarded the genes that, when inactivated, reduced the induction of *irg-1::GFP* by *E. coli* expressing ToxA, reasoning that they were unlikely to be specific regulators of the p38 MAP kinase PMK-1-dependent pathogen response genes. Using this approach, we selected 56 of the 153 genes for further study.

In a tertiary screen, we determined the effects of these 56 gene inactivations on *pF35E12.5::GFP*, a second immune reporter that is also strongly induced in the intestine by RPW-24 in a PMK-1-dependent manner [9], [10]. 29 of the 56 RNAi clones reduced or eliminated the induction of both the *pF35E12.5::GFP* and *pF08G5.6::GFP* reporters by RPW-24 (Table S1A). Validating the screen, the 29 clones we identified as putative regulators of the p38 MAP kinase PMK-1-dependent genes included the three known components of the p38 MAP kinase PMK-1 pathway that were present in the screening library, which suggested that the screen could identify additional, unrecognized components of this signaling pathway.

To confirm further the results of the screen, we used RNAi to knockdown the expression of a representative sample of the 29 genes identified, and tested the induction levels of *F08G5.6* and *F35E12.5* by RPW-24 with qRT-PCR (Table S1A). For all six genes tested, we verified that inactivation of the gene by RNAi dramatically reduced the induction levels of the p38 MAP kinase PMK-1-regulated genes *F08G5.6* and *F35E12.5*.

### Mediator Subunit MDT-15/MED15 Regulates p38 MAP Kinase PMK-1 Pathway-Dependent Genes

One of the strongest hits from our RNAi screen was *mdt-15*, which encodes a subunit of the Mediator complex homologous to mammalian MED15 (78% sequence identity) [18]–[20]. Knockdown of *mdt-15* eliminated all visible expression of the *pF08G5.6::GFP* and *pF35E12.5::GFP* reporters, and reduced expression of these genes by at least two orders of magnitude in response to RPW-24 (Figure 1B and Table S1). MDT-15 was previously found to regulate the transcription of detoxification genes, including cytochrome P450s, glutathione-s-transferases, and UDP-glucuronosyltransferases [19], gene classes that are strongly induced by RPW-24 [9]. To study the role of *mdt-15* in the regulation of p38 MAP kinase PMK-1 gene activation, we crossed the *pF08G5.6::GFP* reporter into the hypomorphic *mdt-15(tm2182)* allele, which was previously shown to recapitulate many of the phenotypes observed in *mdt-15(RNAi)* animals [19], [21]. As in *mdt-15(RNAi)* animals, we observed no induction of GFP when *mdt-15(tm2182); pF08G5.6::GFP* animals were exposed to RPW-24 (Figure 1B). An extrachromosomal array containing wild-type *mdt-15* under its own promoter partially restored RPW-24-induced GFP expression in *mdt-15(tm2182);pF08G5.6::GFP* animals (Figure 1B).

**Figure 2:**
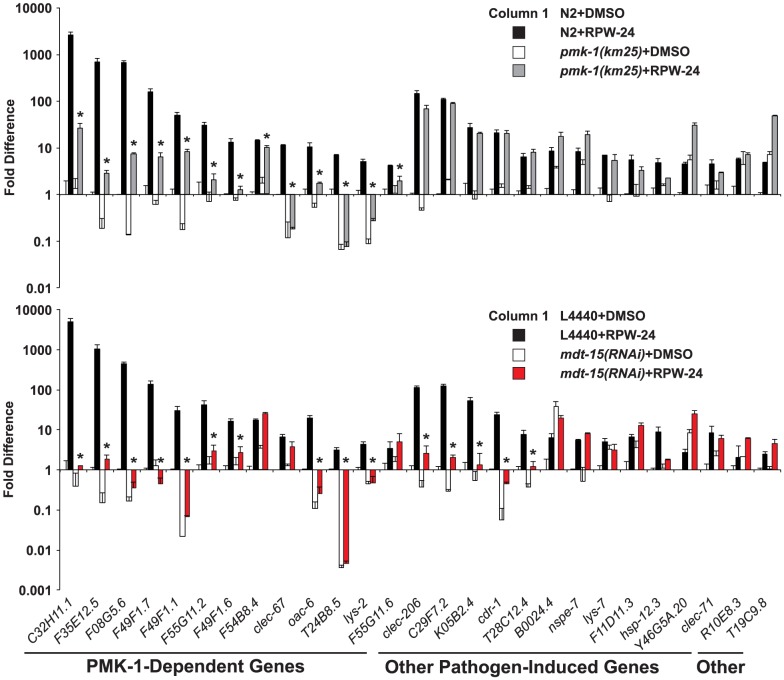
The Mediator subunit MDT-15 regulates p38 MAP kinase PMK-1-dependent and independent immune genes in response to RPW-24. The expression of 118 *C. elegans* genes was analyzed using NanoString nCounter gene expression analysis in wild-type N2 and *pmk-1(km25)* animals (top) and in vector control (L4440) and *mdt-15(RNAi)* animals (bottom) exposed to either 70 µM RPW-24 or the solvent control DMSO. The 40 genes that were induced 4-fold or greater in wild-type N2 animals by RPW-24 are presented in Figures 2 and 6. The 13 genes that were dependent on the p38 MAP kinase PMK-1 for their induction in the top panel are grouped and indicated in the bottom panel. Data are the average of two replicates each of which was normalized to three control genes with error bars representing standard deviation and are presented as the value relative to the average expression from the replicates of the indicated gene in the baseline condition [N2 animals (top) or vector control (L4440) animals (bottom) exposed to DMSO]. We confirmed that *mdt-15* expression was significantly knocked down by *mdt-15(RNAi)* in each of these experiments (*p*<0.001)(see Figure S2A). * *p*<0.05 for the comparison of the RPW-24-induced conditions.

To determine if MDT-15 is required for the induction of other RPW-24-induced genes, we used NanoString nCounter gene expression analysis to generate transcription profiles of 118 *C. elegans* genes with known involvement in immune, stress and detoxification responses (Table S2). As in our microarray analysis [9], we found that RPW-24 caused robust transcriptional changes in wild-type nematodes. 40 of the 118 genes in the NanoString codeset were induced at least 4-fold or greater. 25 of these 40 genes are putative immune effectors upregulated during pathogen infection (shown in Figure 2) and 13 are genes putatively involved in the detoxification of small molecule toxins (discussed below). Of note, we had previously observed that 31 of these 40 genes, including 28 of the 31 most strongly induced, were also upregulated in whole genome Affymetrix GeneChip microarray analysis of wild-type animals exposed to RPW-24 versus DMSO [9]. Of the 25 pathogen-induced genes upregulated by RPW-24, 21 have been shown to be induced during *P. aeruginosa* infection [6], [7], [22], [23]. The RPW-24-induced expression of 13 of the 25 putative immune effectors was significantly reduced in *pmk-1(km25)* loss-of-function mutants compared to wild-type controls (Figure 2 top panel) in accord with the previously determined role for p38 MAP kinase PMK-1 in regulating both pathogen-induced [6] and RPW-24-induced [9] expression of putative immune effector genes.

Consistent with a role for MDT-15 in the expression of PMK-1 activated genes, the NanoString analysis revealed that the RPW-24-dependent induction of 10 of the 13 PMK-1-dependent genes was abrogated in *mdt-15(RNAi)* animals compared to controls (Figure 2 bottom panel). We used qRT-PCR to show that *mdt-15* was knocked down by RNAi in this experiment and to confirm that *mdt-15* depletion caused a dramatic reduction in the RPW-24-induced expression of three *pmk-1*-dependent immune genes (Figure S2A). Further, we found that the RPW-24-mediated induction levels of these three immune genes was reduced in the *mdt-15(tm2182)* mutant, which recapitulated our findings in *mdt-15(RNAi)* animals (Figure S2B).

Knockdown of *mdt-15* reduced the expression of the top five most strongly upregulated p38 MAP kinase PMK-1-dependent immune effectors by several orders of magnitude (*C32H11.1*, *F35E12.5*, *F08G5.6*, *F49F1.7*, and *F49F1.1*) (Figure 2 top panel). These five genes were still induced by RPW-24 in *pmk-1(km25)* loss-of-function animals, albeit to levels markedly lower than in wild-type animals (Figure 2 top panel), but their induction was entirely abrogated by knockdown of *mdt-15* (Figure 2 bottom panel). These data suggest that MDT-15 coordinates inputs from PMK-1 and other immune signaling pathway(s) to modulate the expression of these p38 MAP kinase PMK-1-dependent putative immune effectors. We also identified a requirement for *mdt-15* in the induction of five putative immune effectors (*clec-206*, *C29F7.2*, *K05B2.4*, *cdr-1*, *T28C12.4*) that are not transcriptional targets of the PMK-1 pathway (Figure 2, compare top and bottom panels). Thus, MDT-15 is required for the induction of p38 MAP kinase PMK-1-dependent immune genes and a second group of defense effectors that are independent of the p38 MAP kinase PMK-1 signaling pathway.

**Figure 3:**
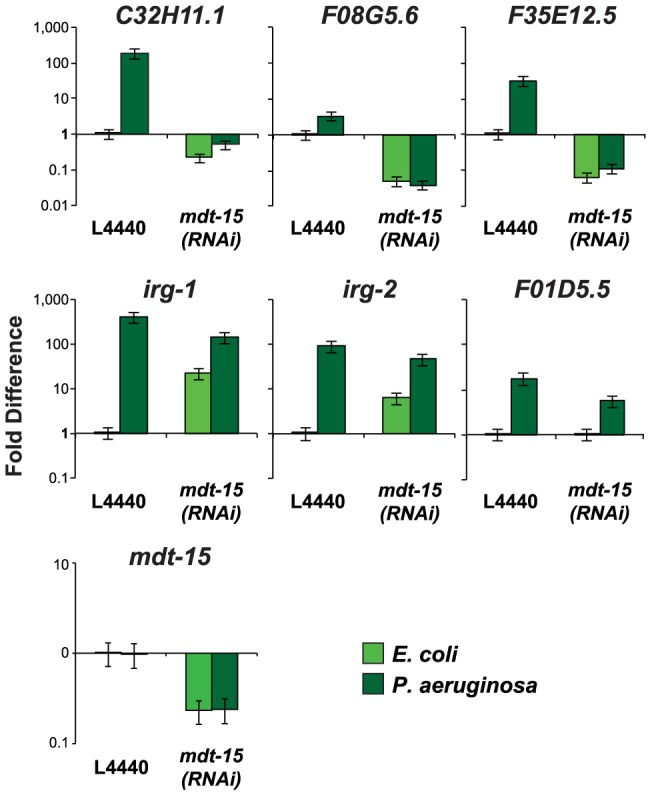
The Mediator subunit MDT-15 regulates the induction of some, but not all, immune genes during *P. aeruginosa* infection. The expression of putative *C. elegans* immune effectors was analyzed by qRT-PCR in vector control (L4440) and *mdt-15(RNAi)* animals exposed to *P. aeruginosa* and the negative control *E. coli* OP50 for 8 hours. Data are the average of three replicates each normalized to a control gene with error bars representing SEM and are presented as the value relative to the average expression from all three replicates of the indicated gene in the baseline condition (L4440 animals exposed to *E. coli*). *mdt-15* expression was significantly knocked down by *mdt-15(RNAi)* in these experiments (*p*<0.001).

The p38 MAP kinase PMK-1 pathway plays an important role in the regulation of putative immune effectors during *P. aeruginosa* infection [6]. To determine whether MDT-15/MED15 is also involved in the regulation of these genes during bacterial infection, we infected *C. elegans* with *P. aeruginosa* PA14 for 8 hours and used qRT-PCR to compare the induction levels of several immune response genes in *mdt-15(RNAi)* and control animals. The basal and pathogen-induced expression of three p38 MAP kinase PMK-1-dependent immune effectors (*C32H11.1*, *F08G5.6* and *F35E12.5*) was reduced in *mdt-15(RNAi)* animals by one to three orders of magnitude (Figure 3). We also tested the induction levels of three genes whose transcription is activated during *P. aeruginosa* infection in a manner independent of PMK-1 (*irg-1, irg-2* and *F01D5.5*) and found that PMK-1-independent immune effectors were induced in *mdt-15(RNAi)* animals during *P. aeruginosa* infection to levels comparable to that observed in wild-type animals (Figure 3). The expression levels of *irg-1* and *irg-2* were higher under basal conditions in *mdt-15(RNAi)* animals compared to L4440 controls (Figure 3). Together, these data support the observations from our NanoString experiments that MDT-15 is required for the expression of some, but not all, immune genes that are activated in response to an environmental insult.

One possible explanation for these observations is that the Mediator subunit MDT-15 is a general regulator of transcription that non-specifically affects the expression of a large number of genes. In the NanoString experiment, however, we identified 10 genes that were induced by RPW-24 in *mdt-15(RNAi)* animals to levels similar to the wild-type controls (Figure 2 bottom panel). Also of note, our secondary screen demonstrated that *mdt-15(RNAi)* had no effect on the induction of *irg-1::GFP* by ToxA. We further wondered if the defects in expression of the PMK-1 targets in *mdt-15(RNAi)* animals were due to direct transcriptional regulation of the p38 MAP kinase PMK-1 pathway components by MDT-15. However, we found that the mRNA levels of *pmk-1*, *tir-1* and *sek-1* in *mdt-15(RNAi)* animals were not different from the wild-type control (Figure S2C).

Taken together, the data in this section indicate that MDT-15 is required for the transcriptional activation of immune effectors controlled by the p38 MAP kinase PMK-1, and at least one other pathway. However, MDT-15 is not required for the induction of all defense-related genes.

### The Mediator Subunit MDT-15 Acts Downstream of the p38 MAP Kinase PMK-1 to Regulate the Induction of *F08G5.6* and *F35E12.5*

**Figure 4:**
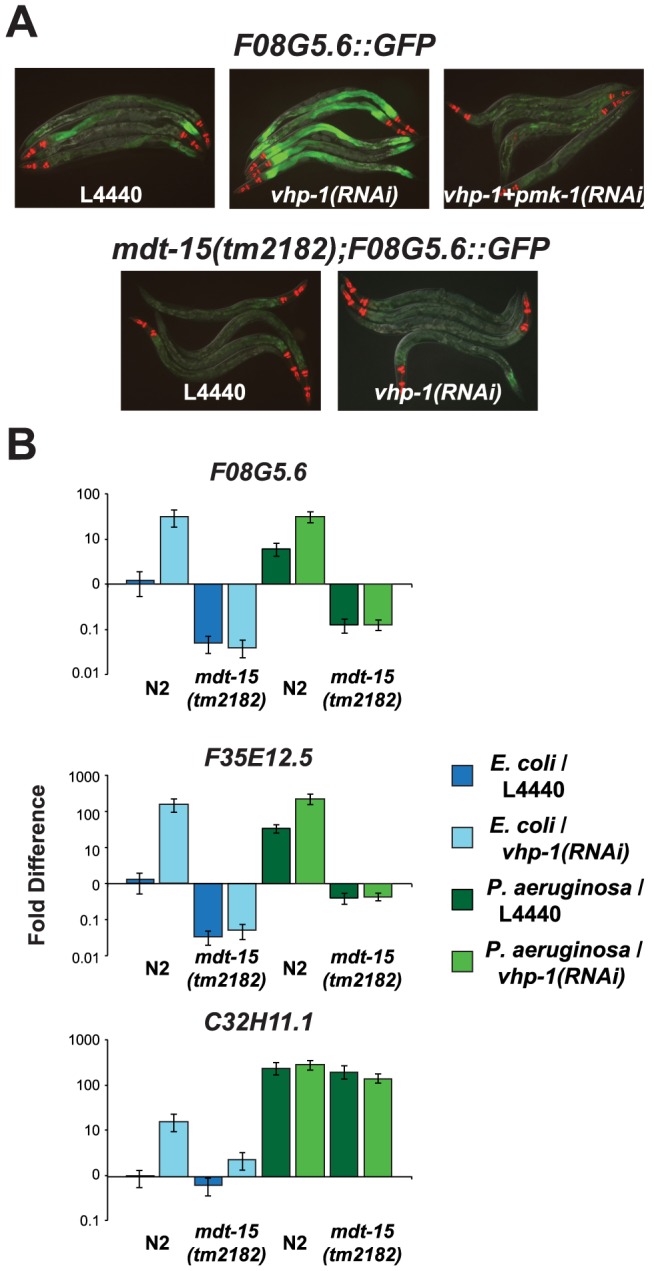
The Mediator subunit MDT-15 acts downstream of the p38 MAP kinase PMK-1 to regulate the induction of *F08G5.6* and *F35E12.5*. (A) Wild-type or *mdt-15(tm2182)* mutant synchronized L1 animals containing the *pF08G5.6::GFP* immune reporter were grown on vector control (L4440), *vhp-1*(RNAi) or a combination of *vhp-1(RNAi)* and *pmk-1(RNAi)* bacteria and then transferred as L4 animals to PA14 for 18 hours. Animals were photographed under the same imaging conditions. (B) qRT-PCR was used to examine the expression levels of *F08G5.6*, *F35E12.5* and *C32H11.1* in wild-type N2 and *mdt-15(tm2182)* mutant animals exposed to *vhp-1(RNAi)* or the vector control (L4440) under basal conditions (as described above) and 8 hours after exposure to *P. aeruginosa*. Knockdown of *vhp-1* caused significant induction of *F08G5.6* and *F35E12.5* in wild-type N2 animals (*p*<0.001), but not in *mdt-15(tm2182)* animals (*p*>0.05), under baseline (*E. coli*) and pathogen-induced conditions. The expression of *C32H11.1* was significantly induced by *vhp-1(RNAi)* (*p*<0.001) in an *mdt-15*-dependent manner under baseline conditions (*p*<0.001), but not following exposure to *P. aeruginosa*. Data are the average of two biological replicates each normalized to a control gene with error bars representing SEM and are presented as the value relative to the average expression of the indicated gene in the baseline condition (L4440 animals exposed to *E. coli*).

To determine if MDT-15 acts genetically upstream or downstream of the p38 MAP kinase PMK-1 in coordinating the induction of immune effectors during *P. aeruginosa* infection, we utilized the MAP kinase phosphatase VHP-1, which is a negative regulator of the p38 MAP kinase PMK-1 [24]. Knockdown of *vhp-1* in animals carrying the *F08G5.6::GFP* p38 MAP kinase PMK-1 immune reporter caused increased induction of GFP expression during *P. aeruginosa* infection in a manner dependent on *pmk-1* expression (Figure 4A). This induction of *F08G5.6::GFP* by *vhp-1(RNAi)* was entirely suppressed in the *mdt-15(tm2182)* partial loss-of-function allele (Figure 4A). We used qRT-PCR to confirm this observation (Figure 4B). We found that RNAi knockdown of *vhp-1* in wild-type *C. elegans* caused constitutive activation of *F08G5.6*, *F35E12.5* and *C32H11.1* in a manner that required *mdt-15* when nematodes were growing on their normal food source, *E. coli* OP50 (Figure 4B). During *P. aeruginosa* infection, we also observed a requirement for MDT-15 in the *vhp-1(RNAi)*-mediated induction of *F08G5.6* and *F35E12.5*, but not *C32H11.1*. The significance of this discrepancy is unclear, but may be explained by the observations of Taubert et al. who found that only 52% of *mdt-15* targets were misregulated in the hypomorphic *mdt-15(tm2182)* allele, which encodes a truncated form of the MDT-15 protein [19], [21]. Indeed, we also observed that *mdt-15(RNAi)* was more effective than *mdt-15(tm2182)* at reducing the expression of the six *mdt-15* targets we studied following exposure to RPW-24, including *C32H11.1* (Figures S2A, S2B and S2D). In any case, these data suggest that MDT-15 acts downstream of the p38 MAP kinase PMK-1 to coordinate the induction of at least some immune effector genes.

### Mediator Subunit MDT-15/MED15 Is Required for Defense against *P. aeruginosa* Infection

**Figure 5:**
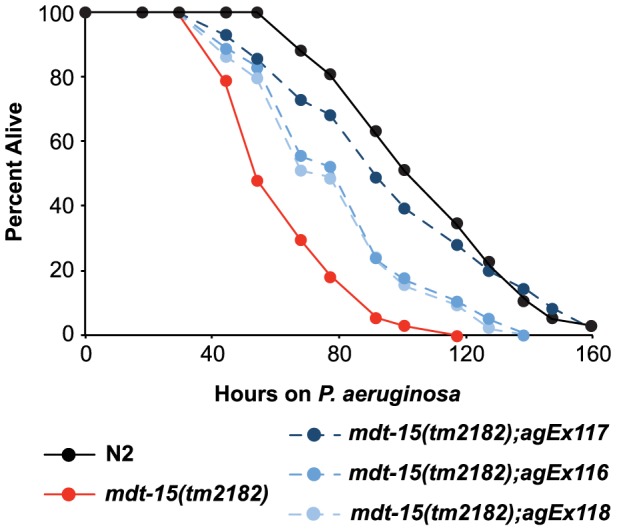
MDT-15 is required for defense against *P. aeruginosa* infection. A *P. aeruginosa* pathogenesis assay with wild-type N2, *mdt-15(tm2182)* mutant worms and *mdt-15(tm2182)* animals carrying *pmdt-15::mdt-15* (three independent lines *agEx116*, *agEx117* and *agEx118*) is shown. The difference in *P. aeruginosa* susceptibility between *mdt-15(tm2182)* animals and each of the three transgenic lines carrying *pmdt-15::mdt-15* is significant, as is the survival difference between N2 and *mdt-15(tm2182)* animals (*p*<0.001). For sample sizes, see Table S3.

The experiments in the preceding sections show that the Mediator subunit MDT-15/MED15 is necessary for the induction of the p38 MAP kinase PMK-1-regulated genes, both in response to RPW-24 and during *P. aeruginosa* infection. We therefore reasoned that mutation or RNAi-mediated knockdown of *mdt-15* would result in enhanced susceptibility to *P. aeruginosa* infection. Initial *P. aeruginosa* pathogenesis assays showed a modest, but significant and reproducible, enhanced susceptibility to infection in *mdt-15(RNAi)* (Figure S3A) and *mdt-15(tm2182)* (Figure S3B) animals compared to controls. However, both *mdt-15(tm2182)* and *mdt-15(RNAi)* animals have reduced brood sizes and varying degrees of sterility. Sterile animals are more resistant to *P. aeruginosa* infection than wild-type animals, due in part to *daf-16*-dependent induction of stress response genes [25]. To eliminate this potentially confounding effect, we made all animals in the *P. aeruginosa* pathogenesis assay sterile by knocking down *cdc-25.1*, a technique that has been used previously in *C. elegans* bacterial pathogenesis assays [26]. Under these conditions, we found that *mdt-15(tm2182)* animals were markedly hypersusceptible to *P. aeruginosa* infection compared to control animals (Figure 5). Moreover, injection of the *mdt-15* gene under control of its own promoter partially rescued the enhanced susceptibility to *P. aeruginosa* phenotype of *mdt-15(tm2182)* animals (Figure 5). Knockdown of *mdt-15* by RNAi also caused a hypersusceptibility to *P. aeruginosa* phenotype in *C. elegans fer-15(b26);fem-1(hc17)* sterile animals [6], [27], and as predicted, to a greater degree than wild-type animals that were not made sterile by *cdc-25.1(RNAi)* (Figure S3C).

We also found that the ability of RPW-24 to extend survival of *P. aeruginosa*-infected wild-type and sterile nematodes was significantly attenuated in *mdt-15(RNAi)* animals compared to controls, suggesting that MDT-15 is required for the immunostimulatory activity of RPW-24 (Figure S3A and S3C). The degree of lifespan extension by RPW-24 during *P. aeruginosa* infection was reduced in *mdt-15(tm2182)* compared to controls, but this difference did not reach statistical significance (*p* = 0.09)(Figure S3B). As discussed above, the gene expression defects of *mdt-15* targets were more severe in *mdt-15(RNAi)* animals than in the hypomorphic *mdt-15(tm2182)* allele [19], [21], which may account for this observation.

One caveat concerning the observation that *mdt-15* depleted animals are hypersusceptible to *P. aeruginosa* infection is that *mdt-15(RNAi)* and *mdt-15(tm2182)* animals have a reduced lifespan when grown on the normal laboratory food source *E. coli* OP50 compared to wild-type controls [18], [19]. Several observations indicate, however, that MDT-15 is an important modulator of nematode survival during bacterial infection. First, we have shown above that *mdt-15(RNAi)* animals fail to upregulate p38 MAP kinase PMK-1-dependent immune effectors both in response to a xenobiotic toxin and during *P. aeruginosa* infection, but retain the ability to induce other immune genes in response to pathogens and following exposure to the bacterial toxin ToxA. In addition, *mdt-15(RNAi)* animals do not respond to the immunostimulatory effects of RPW-24. We have shown previously that *C. elegans* with mutations in the ZIP-1 and FSHR-1 immune pathways, which act in parallel to the p38 MAP kinase PMK-1 cassette, are hypersusceptible to *P. aeruginosa* infection, but retain the ability to respond to RPW-24 [9]. That *mdt-15(RNAi)* animals are blind to the immunostimulatory effects of RPW-24 suggests a specific role of MDT-15 in regulating p38 MAP kinase PMK-1 pathway activity. It is also important to note that despite their reduced lifespan, *mdt-15(RNAi)* animals are not sensitive to all environmental insults. For example, animals deficient in *mdt-15* are sensitive to the toxin fluoranthene, but not β-naphthoflavone, and are not more sensitive to high temperatures than wild-type animals [19].

### Mediator Subunit MDT-15/MED15 Regulates the Induction of p38 MAP Kinase PMK-1-Independent Detoxification Genes and Is Required to Resist the Toxic Effects of the Xenobiotic RPW-24

**Figure 6:**
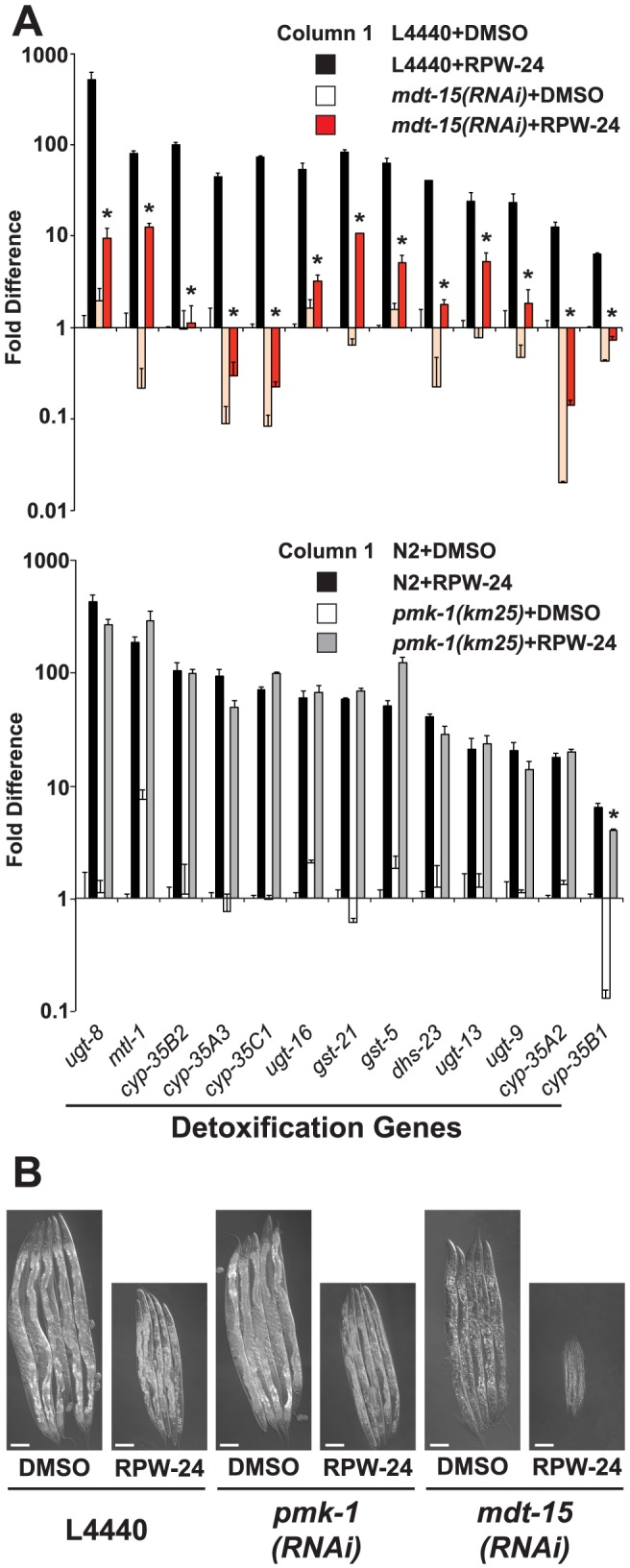
Protection from the toxic effects of the xenobiotic RPW-24 requires MDT-15, but not PMK-1. (A) The thirteen xenobiotic detoxification genes that were induced 4-fold or greater by RPW-24 in the NanoString nCounter gene expression analysis are presented. The top panel compares the RPW-24-mediated induction of these genes in vector control (L4440) and *mdt-15(RNAi)* animals, and the bottom panel shows these data for wild-type N2 versus *pmk-1(km25)* animals, as described in the legend for Figure 2. * *p*<0.05 for the comparison of the RPW-24-induced conditions. (B) Vector control (L4440), *mdt-15(RNAi)* and *pmk-1(RNAi)* animals were exposed to 70 µM RPW-24 or the solvent control DMSO from the L1 stage and photographed after 70 hours of development at 20°C. See Figure S4 for the quantification data from this experiment.

We previously demonstrated that RPW-24 is a xenobiotic toxin [9]. MDT-15 is known to coordinate protection from the toxin fluoranthene and regulate the transcriptional induction of CYPs [19]. We found that the RPW-24-mediated induction of the 13 detoxification genes in the NanoString codeset was nearly entirely abrogated by RNAi knockdown of *mdt-15* (Figure 6A top panel and Table S2). This result was confirmed for three detoxification genes by qRT-PCR (Figures S2B and S2D). To determine if MDT-15 is required to protect *C. elegans* from the toxic effects of RPW-24, we studied the development of wild-type, *pmk-1(RNAi)* and *mdt-15(RNAi)* in the presence of the xenobiotic RPW-24, an assay that has been used previously to assess the toxicity of small molecules [19]. We found that RPW-24 slowed the development of control and *pmk-1(RNAi)* animals to similar degree, and that *mdt-15(RNAi)* animals were markedly delayed in the presence of RPW-24 (Figures 6B and S4). These data show that MDT-15 controls the induction of detoxification genes following exposure to RPW-24 and is required to resist the toxic effects of this xenobiotic.

Given that MDT-15 is required for the regulation of detoxification genes and the p38 MAP kinase PMK-1 pathway, we wondered if the detoxification machinery in *C. elegans* is regulated by the p38 MAP kinase PMK-1. We found, however, that 12 of the 13 RPW-24-induced detoxification genes were upregulated in *pmk-1(km25)* null mutants to levels comparable to that in wild-type animals exposed to RPW-24 (Figure 6A bottom panel). We used qRT-PCR to confirm this observation for three cytochrome P450 genes (Figure S2D). Thus, the MDT-15-dependent xenobiotic detoxification program is induced in a manner independent of the p38 MAP kinase PMK-1.

### MDT-15/MED15 Is Not Required for the Avoidance Behavior Induced by RPW-24

Many xenobiotic toxins, including RPW-24, induce an avoidance response wherein *C. elegans* leave a lawn of bacterial food, to which they are otherwise attracted, if it contains a toxic compound [3], [9]. We therefore wondered if MDT-15 is required for the avoidance behavior induced by RPW-24. However, *pmk-1(km25)* and *mdt-15(tm2182)* animals left the lawn of *E. coli* containing RPW-24 as readily as wild-type animals (Figure S5). We also observed a similar phenotype when we knocked down the expression of *pmk-1* and *mdt-15* in the neuronally-sensitive RNAi strain TU3311 (data not shown). Thus, animals lacking the function of MDT-15 and PMK-1 are still able to recognize RPW-24 as a toxin.

In summary, the data in this and the preceding section suggest that MDT-15, but not PMK-1, controls the induction of genes that are required to resist the toxic effects of RPW-24, and that neither are required for avoidance of RPW-24.

### Mediator Subunit MDT-15/MED15 Promotes Resistance to Secreted *P. aeruginosa* Phenazine Toxins

**Figure 7:**
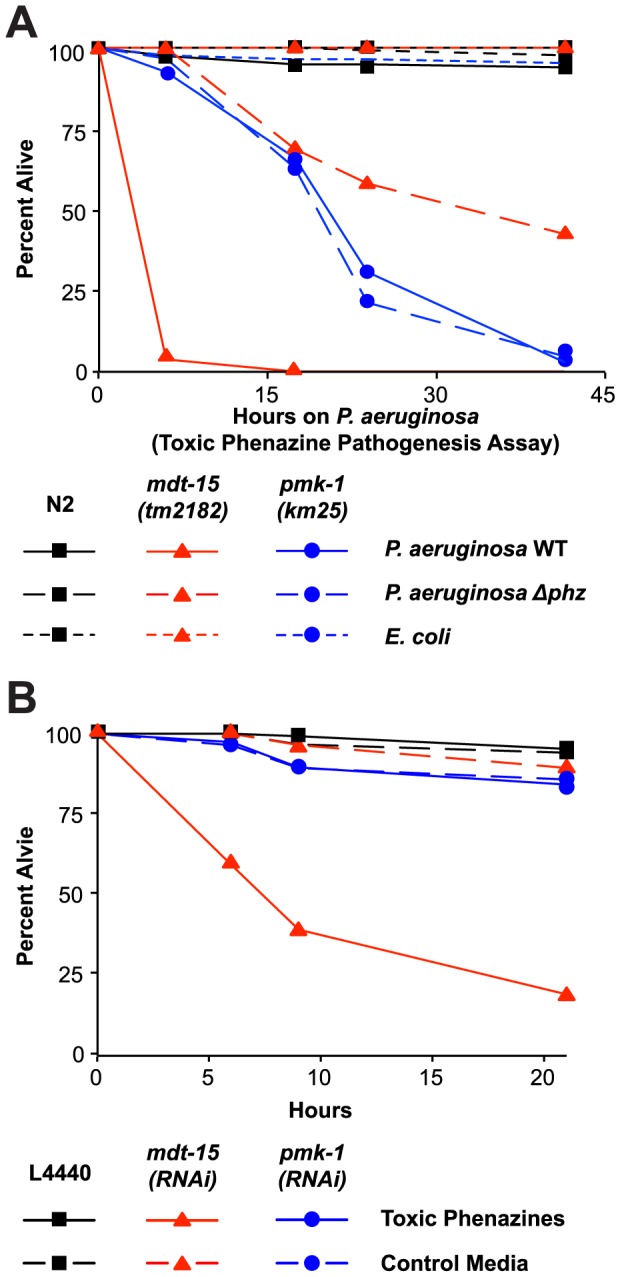
Resistance to *P. aeruginosa* phenazine toxins requires MDT-15. (A) Wild-type N2, *mdt-15(tm2182)* and *pmk-1(km25)* animals were tested in the toxic phenazine *P. aeruginosa* pathogenesis assay (also called “fast killing” assay). *C. elegans* young adult animals were exposed to wild-type (WT) *P. aeruginosa*, *P. aeruginosa* carrying a deletion in both of the phenazine biosynthetic operons (*Δphz*) or *E. coli* OP50. The difference between *mdt-15(tm2182)* and N2 animals exposed to *P. aeruginosa* WT is significant (*p*<0.001), as is the difference in *mdt-15(tm2182)* animals exposed to *P. aeruginosa* WT and *P. aeruginosa Δphz (p*<0.001). There is no significant difference in *pmk-1(km25)* animals exposed to *P. aeruginosa* WT and *P. aeruginosa Δphz* (*p*>0.05). This assay is representative of two independent experiments. Figure S6A presents the control condition for this experiment showing that both *C. elegans* N2 and *mdt-15(tm2182)* at the L4 stage are sensitive to toxic phenazine-mediated killing (*p*<0.001). (B) Vector control (L4440), *mdt-15(RNAi)* and *pmk-1(RNAi)* animals were exposed to high osmolarity “fast kill” media (pH 5) with *E. coli* as the food source in the presence or absence of phenazine-1-carboxylic acid and 1-hydroxyphenazine (labeled “toxic phenazines” above) at the approximate concentrations produced by *P. aeruginosa* under standard assay conditions. The difference in killing between *mdt-15(RNAi)* animals exposed to toxic phenazines and the other conditions is significant (*p*<0.001). See also Figure S6. For sample sizes, see Table S3.

We have shown that MDT-15 regulates the *C. elegans* detoxification response to the xenobiotic RPW-24. We therefore hypothesized that MDT-15 might also be required for protection from lethal secreted toxins produced by pathogenic bacteria. To address this question, we used an assay that allows the specific study of *C. elegans* killing by secreted low molecular weight toxins of *P. aeruginosa*. When *P. aeruginosa* is grown on high osmolarity media, phenazine toxins produced by the bacteria are lethal to nematodes [28], [29]. In contrast to the “slow killing” infection assay, which was used in the assays described above, wild-type nematodes exposed to these “fast killing” conditions die within a few hours via a process that does not require live bacteria [28], [29]. Wild-type *C. elegans* at the fourth larval stage of development, but not young adult animals, are exquisitely sensitive to the phenazine toxins produced by *P. aeruginosa* in this assay and are killed within 6 hours of exposure. We took advantage of the inherent resistance of young adult animals to test whether *mdt-15(tm2182)* animals are hypersusceptible to the phenazine toxins produced by *P. aeruginosa*. As expected, both wild-type and *mdt-15(tm2182)* L4 animals were rapidly killed by *P. aeruginosa* in the “fast kill” assay (Figure S6A). *P. aeruginosa* carrying deletions of both phenazine biosynthetic operons (*Δphz*) does not make these toxins and accordingly had reduced ability to kill both wild-type and *mdt-15(tm2182)* animals (Figure S6A). In contrast to L4 animals, we observed that almost no young adult, wild-type animals were killed after six hours of exposure to the phenazine toxins compared with 98% death of L4 staged animals (Figures 7A and S6A), which reproduces the findings of others [28], [29]. In contrast to wild-type animals, young adult *mdt-15(tm2182)* animals were dramatically susceptible to *P. aeruginosa* in this assay and this pathogenesis required the secretion of phenazine toxins, as *P. aeruginosa Δphz* was markedly less pathogenic toward *mdt-15(tm2182)* young adults (Figure 7A). Moreover, *mdt-15(tm2182)* animals were not simply hypersusceptible to the high osmolarity conditions of this assay because we observed no mortality over the course of the assay in *mdt-15(tm2182)* mutants exposed to “fast kill” media containing the normal nematode food source *E. coli* OP50 (Figure 7A).

We confirmed that phenazine toxins are lethal to *mdt-15*-depleted animals by supplementing “fast kill” growth media with both phenazine-1-carboxylic acid and 1-hydroxyphenazine in the absence of pathogen [28]. These two particular phenazines are toxic to wild-type nematodes [28] and, as expected, the mixture of phenazine-1-carboxylic acid and 1-hydroxyphenazine rapidly killed L4 animals (Figure S6B). Young adult wild-type animals were resistant to the lethal effects of these molecules (Figure 7B). However, *mdt-15(RNAi)* young adult animals were dramatically susceptible to phenazine-mediated killing (Figure 7B).

We also found that *pmk-1(km25)* loss-of-function mutants were more susceptible to *P. aeruginosa* in the “fast killing” assay than wild-type animals, although were less susceptible than *mdt-15(tm2182)* animals. This lethality, however, was mediated by factors other than phenazine toxins, since there was no difference in pathogenicity of the wild-type and *Δphz P. aeruginosa* toward *pmk-1(km25)* mutants (Figure 7A). Likewise, *pmk-1(RNAi)* animals were not more susceptible to phenazines-1-carboxylic acid and 1-hydroxyphenazine than L4440 RNAi control animals (Figure 7B).

Together, these data define a role for MDT-15, but not PMK-1, in the protection from bacterial-derived phenazine toxins.

## Discussion

Detecting and countering environmental threats is central to the ability of organisms to survive and reproduce in the wild. We examined the *C. elegans* response to the xenobiotic RPW-24, which is able to induce a host immune response that is protective for animals infected with the lethal bacterial pathogen *P. aeruginosa* [9]. In an RNAi screen with RPW-24, we identified a number of genes, including *mdt-15/MED15*, which are required for induction of p38 MAP kinase PMK-1-dependent immune effectors. *mdt-15* encodes a subunit of the highly conserved Mediator complex that controls the activation of a variety of genes involved in the response to external stress. We demonstrate that: *(i)* MDT-15 is required for the induction of p38 MAP kinase PMK-1-dependent immune effectors following exposure to a xenobiotic toxin, as well as during infection with *P. aeruginosa*, *(ii)* MDT-15 controls the expression of some p38 MAP kinase PMK-1-independent immune effectors, but not all defense genes, (*iii*), MDT-15 functions downstream of the p38 MAP kinase PMK-1 cascade to control the induction of at least two immune effectors, *(iv)* the induction of xenobiotic detoxification genes and protection from the toxic effects of RPW-24 requires MDT-15, but not the p38 MAP kinase PMK-1, and (*iv*) MDT-15 is necessary for protection during *P. aeruginosa* infection and from phenazine toxins secreted by this organism.

The Mediator complex is strongly conserved from yeasts to humans, and is required for transcription by physically interacting with and directing the activity of RNA polymerase II [30], [31]. Although initial studies from yeast described the Mediator complex as a general regulator of transcription [32], it is now becoming clear that the individual subunits of this complex, of which there are at least 26, play important roles in translating inputs from cell signaling pathways to specific outputs [30], [31], [33]. For example, the regulation of chemotherapeutic resistance in cancer cells requires MED12 [33], MED23 channels MAP kinase signaling activity to coordinate cell growth [34], and MED15 serves as a regulatory node in lipid metabolism by directing the activity of the transcriptional activator SREBP (sterol regulatory element binding protein) [20]. Interestingly, MED15's role as a lipid sensor is strongly conserved. In *C. elegans*, the MED15 homolog MDT-15 interacts with SBP-1, the SREBP homolog, and serves as an important regulator of lipid metabolism [18]–[20]. Likewise, the MED15 homolog in yeast (called GAL11p) serves a similar function [35].

Taubert et al. showed in a *C. elegans* genome-wide microarray analysis that MDT-15 coordinates the transcription of Phase I and Phase II detoxification genes, and is required to resist the lethal effects of a xenobiotic toxin [19] and agents that induce oxidative stress [21]. Consistent with these observations, we found that MDT-15 is necessary to mount a detoxification response toward and provide protection from RPW-24, a xenobiotic toxin. Our studies extend the known functions of this conserved Mediator subunit to innate immunity in demonstrating that MDT-15 plays a critical role in controlling immune and detoxification pathway activation in a manner that is important for protection from bacterial infection and from the secreted phenazine toxins of *P. aeruginosa*.

We propose that the Mediator subunit MDT-15 links innate immune activation to xenobiotic detoxification responses as part of a general strategy to ensure survival in harsh environments. In their natural habits, nematodes encounter numerous threats from ingested pathogens and xenobiotic toxins [1]. Thus, coupling xenobiotic detoxification to innate immune activation may have been selected for by pathogens that secrete soluble toxins during infection. Whether the protection from phenazine toxins mediated by MDT-15 requires detoxification genes or occurs via another mechanism is not known, but it is interesting to note that MDT-15's function as a regulator of host protection may be evolutionarily conserved. In yeasts, the MDT-15 homolog Gal11p coordinates a protective cellular response following xenobiotic exposure, which is manifest as the upregulation of drug efflux pumps [36].

The data presented in this study imply the existence of surveillance mechanisms that monitor for xenobiotic toxins (or their effects) and signal through MDT-15/MED15 to simultaneously activate protective detoxification and innate immune responses. Whether such activation occurs in the context of xenobiotic-induced organelle dysfunction is not known. McEwan et al. [15] and Dunbar et al. [16] found that *C. elegans* monitor the integrity of its translational machinery as a means to detect pathogen invasion. Inhibition of translation by the secreted bacterial toxin ToxA leads to an antibacterial response via known immune pathways involving the transcription factor ZIP-2 and the p38 MAP kinase PMK-1. Several observations suggest, however, that MDT-15-mediated regulation of immune activation and detoxification gene induction by RPW-24 does not occur via a mechanism involving translation inhibition. First, we previously found that a loss-of-function mutation in the *zip-2* gene did not affect the ability of RPW-24 to extend the survival of animals infected with *P. aeruginosa* and genome-wide transcriptional analysis of animals exposed to RPW-24 did not suggest that this compound is an inhibitor of translation [9]. Second, the transcriptome analysis of animals exposed to ToxA did not show an abundance of Phase I and II detoxification genes [15]. Finally, we show here that the ToxA-responsive, ZIP-2-regulated genes, *irg-1* and *irg-2*, are induced in *mdt-15(RNAi)* animals during *P. aeruginosa* infection to levels comparable to that observed in wild-type animals.

Melo et al. recently studied the behavioral response of *C. elegans* to xenobiotic toxins known to disrupt the function of the mitochondria, the ribosome, and the endoplasmic reticulum [3]. Inhibiting these essential processes by the action of small molecules or through targeted gene disruptions triggered an avoidance response, which required serotonergic and JNK signaling pathways. They proposed that organisms monitor disruption of core metabolic processes as a means to detect pathogen invasion and challenges from xenobiotic toxins. Based on the data reported here, however, it is not clear, whether immune signaling is an integral part of xenobiotic-elicited avoidance behavior, at least with respect to RPW-24. We found that the function of neither MDT-15 nor PMK-1 was required for the avoidance of RPW-24, indicating that the behavioral component of this protective response occurs upstream of MDT-15, or via separate mechanism altogether.

It will be interesting to determine the mechanism by which MDT-15 activates immune and detoxification responses in *C. elegans*. In our genetic screen for regulators of the p38 MAP kinase PMK-1 pathway, we identified a number of genes that are involved in fatty acid biosynthesis, including *mdt-15, fat-6, fat-7, elo-5, acs-19* and *C25A1.5*. Indeed, MDT-15 is known to control the expression of *fat-6* and *fat-7* [18]–[20]. It is therefore possible that a fatty acid signaling molecule or membrane component is required for p38 MAP kinase PMK-1 activity. We found, however, in epistasis analyses with the MAP kinase phosphatase VHP-1 that MDT-15 functions downstream of PMK-1 to coordinate the expression of *F08G5.6* and *F35E12.5*. Thus, for at least a subset of immune genes, MDT-15 likely also physically interacts with sequence-specific regulators, such as ATF-7, a transcription factor that is the downstream signaling target of the p38 MAP kinase PMK-1 pathway [12], to coordinate protective host responses mounted following exposure to xenobiotic toxins.

## Materials and Methods

### *C. elegans* and Bacterial Strains

*C. elegans* were grown on standard NGM plates with *E. coli* OP50 [37] unless otherwise noted. The previously published *C. elegans* strains used in this study were: N2 Bristol [37], *pmk-1(km25)* [5], AY101 [*acIs101*[*pDB09.1(pF35E12.5::GFP*); pRF4(*rol-6(su1006))*] [10], XA7702 *mdt-15(tm2182)* [19], [21], CF512 *fer-15(b26);fem-1(hc17)* [38], and AU0133 [*agIs17*(*pirg-1::GFP*; *pmyo-2::mCherry)*] [14]. The *C. elegans* strains created for this study were: AU0307 [*agIs44(pF08G5.6::GFP::unc-54-3′UTR; pmyo-2::mCherry)*], AU0316 [*mdt-15(tm2182); agIs44*], AU0325 [*mdt-15(tm2182); agEx116 (mdt-15;pmyo-3::mCherry*)], AU0326 [*mdt-15(tm2182); agEx117 (mdt-15;pmyo-3::mCherry)*], AU0327 [*mdt-15(tm2182); agEx118 (mdt-15;pmyo-3::mCherry)*] and AU0323 [*mdt-15(tm2182); agIs44; agEx114 (mdt-15;pmyo-3::mCherry)*].

The strain carrying *agIs44* was constructed by PCR amplification from N2 genomic DNA of an 851 bp region upstream of the start codon of the *F08G5.6* gene (primers GACTTGTCAAATGAACAATTTTATCAAATCTCA and CGCCTAGGTGTCAATTGATAATGAATA) and ligated to the GFP coding region and *unc-54-3′UTR* sequences amplified from pPD95.75 using published primers, and a previously described protocol [39]. The *agIs44* construct was transformed into N2 animals with the co-injection marker *pmyo-2::mCherry* using established methods [40]. A strain carrying the *pF08G5.6::GFP::unc-54-3′UTR* and *pmyo-2::mCherry* transgenes in an extrachromosomal array was irradiated, and strains carrying the integrated array *agIs44* were isolated. AU0307 was backcrossed to N2 five times.

The *mdt-15* rescuing arrays agEx116, agEx117 and agEx118 contain a 4.8 kb *mdt-15* genomic fragment, which includes 707 bp upstream and 1075 bp downstream of the *mdt-15* coding region, amplified from N2 genomic DNA (primers GGAGTATCAGAAGCTCACGATGCTC and CCAAATAATACTAACCACCACATATCTTCCATT). This *mdt-15* genomic fragment was transformed into N2 animals or AU0316 with the co-injection marker *pmyo-3::mCherry* using established methods.

RNAi clones presented in this study were from the Ahringer [41] or Vidal [42] RNAi libraries unless otherwise stated. The *atf-7* [12] and the *pmk-1* [5] RNAi clones have been previously reported. All RNAi clones presented in this study have been confirmed by sequencing. The *P. aeruginosa* strain PA14 were used for all studies, unless otherwise indicated. The *P. aeruginosa* strains used in Figure S1 have been previously described [13] and were (in order of descending virulence toward *C. elegans*): CF18, PA14, MSH10, S54485, PA01, PAK, 19660, and E2. The *P. aeruginosa* PA14 phenazine null mutant (*Δphz*) lacks both the *phzA1-G1* and *phzA2-G2* operons and has been previously described [43]. The BL21 *E. coli* strain that expresses the bacterial toxin Exotoxin A (ToxA) has been previously described [15].

### Feeding RNAi Screen

1,420 RNAi clones that correspond to genes expressed in the *C. elegans* intestine based on their annotation in Wormbase (www.wormbase.org) in April, 2008 were selected from the Ahringer [41] or Vidal [42] RNAi libraries (see Table S1B). RNAi clones were pinned into 1.2 ml of LB plus 100 µg/ml carbenicillin in 96-well culture blocks (Corning Incorporated) and grown overnight at 37°C with shaking at 950 RPM in a Multitron II Shaking Incubator (Appropriate Technical Resources). 40 µL of the 10× concentrated overnight culture were added to each well of a 24-well plate containing RNAi agar medium and grown overnight at room temperature. The following day, 50–100 L1 staged AU0307 animals, which carry the *agIs44* transgene, were added to each well and allowed to grow until they were at the L4 or young adult stage. Worms were then transferred to new 24-well screening plates containing 1 mL of “slow kill” media supplemented with 70 µM RPW-24 and seeded with *E. coli* OP50 food. Animals were dried on the screening plates for several hours at room temperature and then incubated overnight at 20C. The L4440 vector and *pmk-1* RNAi clones were included on each of the screening plates as the negative and positive controls, respectively. Animals were scored for GFP expression and rated on a subjective scale from 0 (no GFP expression in response to RPW-24) to 3 (RPW-24-mediated induction of GFP expression equivalent to L4440). Exposure of the *C. elegans* transcriptional reporter *irg-1::GFP* to an *E. coli* strain that expresses ToxA was performed as previously described [15].

### *C. elegans* Bacterial Infection and Other Assays

“Slow killing” *P. aeruginosa* infection were performed as previously described [9], [44]. In all of these assays, the final concentration of DMSO was 1% and RPW-24 was used at a concentration of 70 µM, unless otherwise indicated. The propensity of wild-type *C. elegans* to leave a lawn of bacteria supplemented with RPW-24 was assayed using a previously described protocol [3], [9] with minor modifications. Rather than adding the toxin on top of the small lawn of food, 20 µg of RPW-24 was mixed with *E. coli* OP50, which was spotted onto NGM plates. To assess the toxicity of RPW-24, we assayed the development of animals exposed to vector control (L4440), *pmk-1(RNAi)* and *mdt-15(RNAi)* in the presence of 70 µM RPW-24, as previously described [9]. *P. aeruginosa* “fast kill” pathogenesis assays were conducted with late L4 and early young adult animals (picked 1–3 hours after the L4 molt) obtained from timed egg lays as described [28], [29]. For the killing assay using toxic phenazines, 50 µg/ml phenazine-1-carboxylic acid (PCA) and 5 µg/ml 1-hydroxyphenazine in DMSO were added to modified “fast kill” media (1% bacto-peptone, 1% glucose, 1% NaCl, 150 mM sorbitol, 1.7% bacto agar, 5 µg/ml cholesterol and 50 mM sodium citrate, pH 5) [28]. These phenazine concentrations correspond to the amount of PCA and 1-hydroxy-phenazine that are produced under “fast kill” conditions [28]. *E. coli* OP50 was used as the food source. The modified “fast kill” media pH 5.0 plus 1% DMSO was used as the control condition. These assays were incubated at 21–23°C.

### Quantitative RT-PCR (qRT-PCR) and NanoString nCounter Gene Expression Analyses

Synchronized L1 staged *C. elegans* N2 animals were grown to L4/young adult stage on the indicated RNAi strain, transferred to assay plates and incubated at 25°C for 24 hours. To prepare the assay plates, 70 µM RPW-24 or DMSO was added to 20 mL “slow killing” media [44] in 10 cm petri dishes seeded with *E. coli* OP50. N2 and *pmk-1(km25)* animals were raised on *E. coli* OP50 and exposed to the above conditions for 18 hours at 20°C. For qRT-PCR studies of nematodes infected with *P. aeruginosa* PA14 or the indicated strain of *P. aeruginosa*, 20 mL of “slow killing” media containing either DMSO or 70 µM RPW-24 was added to 10 cm petri dishes. Plates were seeded with either 75 µL of *E. coli* OP50 or *P. aeruginosa*, each from cultures grown for 15 hours at 37°C. The plates were incubated for 24 hours at 37°C and 24 hours at 25°C. L4/young adult animals were added to the assay plates and incubated at 25°C for eight hours. RNA was isolated using TriReagent (Molecular Research Center, Inc.) and analyzed by NanoString nCounter Gene Expression Analysis (NanoString Technologies) using a “codeset” designed by NanoString that contained probes for 118 *C. elegans* genes (Table S2). Probe hybridization, data acquisition and analysis were performed according to instructions from NanoString with each RNA sample normalized to the control genes *snb-1*, *ama-1* and *act-1*. For the qRT-PCR studes, RNA was reverse transcribed to cDNA using the Retroscript kit (Life Technologies) and analyzed using a CFX1000 machine (Bio-Rad) with previously published primers [6], [18]. The qRT-PCR primers for the *mdt-15*, *pmk-1*, *tir-1* and *sek-1* genes were designed for this study and are available upon request. All values were normalized against the control gene *snb-1*. Fold change was calculated using the Pfaffl method [45].

### Microscopy

Nematodes were mounted onto agar pads, paralyzed with 10 mM levamisole (Sigma) and photographed using a Zeiss AXIO Imager Z1 microscope with a Zeiss AxioCam HRm camera and Axiovision 4.6 (Zeiss) software. For comparisons of GFP expression in the *F08G5.6::GFP* transgenic animals, photographs were acquired using the same imaging conditions.

### Statistical Analyses

Differences in survival of *C. elegans* animals in the *P. aeruginosa* pathogenesis assays were determined with the log-rank test in each of two biological replicates. Differences were considered significant only if the *p* value was less than 0.05 for both replicates. In the manuscript, data from one experiement that is representative of both replicates is shown and the sample sizes for these experiments are given in Table S3. To determine if the increase in survival conferred by RPW-24 treatment was different in one population compared to another, we examined the difference in the effect of RPW-24 treatment on the hazard in each group using a Cox proportional hazard model (Stata13, Stata, College Station, TX) from two biological replicates, as previously described [9]. Fold changes in the qRT-PCR analyses were compared using unpaired, two-tailed student *t*-tests.

### Accession Numbers

Accession numbers for genes and gene products are given for the publically available database Wormbase (http://www.wormbase.org). The accession numbers for the principal genes mentioned in this paper are: *atf-7 (C07G2.2), C32H11.1, cyp-35A1 (C03G6.14), cyp-35B2 (K07C6.3), cyp-35C1 (C06B3.3), F35E12.5, F08G5.6, F01D5.5, fshr-1 (C50H2.1), irg-1 (C07G3.2), irg-2 (C49G7.5), mdt-15 (R12B2.5), nsy-1 (F59A6.1), pmk-1 (B0218.3), sek-1 (R03G5.2), skn-1 (T19E7.2), tir-1(F13B10.1)*, and *zip-2 (K02F3.4)*. Other accession numbers are given in Figure 2, Figure 6, Table S1 and Table S2.

## Supporting Information

Figure S1 ***P. aeruginosa*** **strains with greater virulence towards** ***C. elegans*** **cause more robust induction of the immune response gene** ***F08G5.6*** **.** (A) Wild-type N2 *C. elegans* were exposed for eight hours to eight *P. aeruginosa* strains, each with a different virulence potential toward *C. elegans* [13]. Data are the average of two biological replicates, each normalized to a control gene with error bars representing SEM and are presented as the fold induction of *F08G5.6* compared to its expression in *C. elegans* exposed to *E. coli* OP50. *p<0.05 for the difference from the *F08G5.6* induction in the most virulent *P. aeruginosa* strain (CF18). (B) Worms carrying the *pF08G5.6::GFP* reporter were exposed to *P. aeruginosa* PA14 and PAK for 18 hours, and photographed under the same conditions.
(EPS)

Figure S2 **The Mediator subunit MDT-15 regulates immune and detoxification genes in response to RPW-24.** (A) The expression of putative *C. elegans* immune effector genes was analyzed by qRT-PCR in vector control (L4440) and *mdt-15(RNAi)* animals exposed to either 70 µM RPW-24 or the solvent control DMSO, as indicated. The difference in expression of each of the genes in both *mdt-15(RNAi)* conditions was significantly different from vector control (L4440) (*p*<0.001). (B) The RPW-24-induced expression levels for the indicated genes were compared in *mdt-15(tm2182)* mutant animals and wild-type controls. Data are presented as the average expression value of the indicated gene following RPW-24 exposure in *mdt-15(tm2812)* animals versus wild-type animals. (C) The expression levels of three components of the p38 MAP kinase PMK-1 pathway were compared in animals exposed to 70 µM RPW-24 or DMSO. (D) RPW-24-mediated induction levels of *cyp-35B1*, *cyp-35B2* and *cyp-35C1* were assessed in *pmk-1(km25)* and wild-type N2 animals as described above. There was no significant difference in the induction of these genes (*p*>0.05). All data in this figure are the average of two or three replicates, each normalized to a control gene with error bars representing SEM. For the analyses in A and C, data are presented as the value relative to the average expression from all replicates of the indicated gene in the baseline condition [L4440 (A) or N2 (C) animals exposed to DMSO].
(EPS)

Figure S3 **The Mediator subunit MDT-15 is required for defense against** ***P. aeruginosa*** **infection.** *C. elegans* wild-type N2 (A) and *fer-15(b26);fem-1(hc17)* sterile (C) animals were exposed to vector control (L4440) or *mdt-15(RNAi)* and infected with *P. aeruginosa* in the presence of the solvent control DMSO or 70 µM RPW-24. There is a significant difference in susceptibility to *P. aeruginosa* infection between the L4440/DMSO and *mdt-15(RNAi)*/DMSO conditions in A and C (*p*<0.001). In addition, the magnitude of lifespan prolongation conferred by RPW-24 during *P. aeruginosa* infection was significantly reduced in *mdt-15(RNAi)* animals compared to control animals for both assays (*p*<0.001). (B) *C. elegans* wild-type N2 or *mdt-15(tm2182)* animals were infected with *P. aeruginosa* in the presence of the solvent control DMSO or 70 µM RPW-24. There is a significant difference between the N2/DMSO and *mdt-15(tm2182)*/DMSO conditions, which was also significant in a biological replicate of this experiment (*p*<0.001). The degree of lifespan extension conferred by RPW-24 exposure during *P. aeruginosa* infection was reduced in *mdt-15(tm2182)* animals compared to wild-type animals, but the difference did not reach statistical significance (*p* = 0.09). In A, B and C, data at each time point are the average of three plates per strain, each with approximately 30–40 animals per plate (see Table S3 for sample sizes). Each assay presented in this figure is representative of two independent experiments.
(EPS)

Figure S4 **Protection from the toxic effects of the xenobiotic RPW-24 requires MDT-15, but not PMK-1.** The percentage of the population of animals that were at the indicated development stage after 70 hours of incubation at 20°C is presented. Adult animals all had a fully formed vulva and were divided into three categories based on the number of eggs carried in the animal. Larval stages were determined based on microscopic examination of the gonad. See Table S3 for the sample sizes.
(EPS)

Figure S5 **Neither MDT-15 nor PMK-1 are required for the behavioral avoidance response to RPW-24.** (A) The percentage of wild-type N2, *pmk-1(km25)* and *mdt-15(tm2182)* animals that were off a lawn of *E. coli* OP50 supplemented with either DMSO or 20 µg RPW-24 (average of three replicates) 16 hours after exposure is presented with error bars representing standard deviation. There is no significant difference in the percentage of animals off a lawn containing RPW-24 among the conditions in this experiment (*p*>0.05). See Table S3 for the sample sizes. (B) Representative photographs of each condition are shown.
(EPS)

Figure S6 **Resistance to** ***P. aeruginosa*** **phenazine toxins requires MDT-15.** The controls for the experiment in Figure 7 are presented. (A) *C. elegans* wild-type N2 and *mdt-15(tm2182)* animals at the L4 stage are sensitive to killing by *P. aeruginosa* in the “fast killing” toxic phenazine assay. (B) Likewise, L4 staged nematodes are rapidly killed by toxic phenazines added to assay media in the absence of pathogen. These experiments were conducted in parallel with and as described for the data presented in Figure 7.
(EPS)

Table S1 **Genes identified in an RNAi screen for regulators of the p38 MAP kinase PMK-1 pathway.** (A) Presented are the 29 genes that were identified in a screen of 1,420 RNAi clones, the complete list of which is given (B), because they reduce or abrogate the induction of the p38 MAP kinase PMK-1-dependent immune reporters *F08G5.6::GFP* and *F35E12.5::GFP*. Knockdown of these genes does not affect the induction of *irg-1::GFP* by an *E. coli* strain engineered to express ToxA. qRT-PCR of three replicates was used to confirm that the RNAi-mediated knockdown of the indicated gene in wild-type animals reduced the induction of *F08G5.6* and *F35E12.5* by RPW-24. We previously showed that *atf-7* and *pmk-1* are required for the RPW-24-mediated induction of *F08G5.6* and *F35E12.5* [9]. These 29 genes were identified in an RNAi screen of 1,420 clones, which are presented in (B). Of note, the 29 genes presented in (A) were sequenced confirmed.
(XLSX)

Table S2 **Relative expression of the 118 genes in the nCounter Gene Expression Analysis.** Nanostring nCoutner Gene Expression Analysis data for (A) N2 wild-type and *pmk-(km25)*, and (B) Vector control (L4440) and *mdt-15(RNAi)*, each exposed to DMSO or RPW-24, are presented. Expression values are the average of two independent replicates each of which was normalized to three control genes and are presented as the value relative to the average expression of the indicated gene in the baseline condition [N2 animals (A) or vector control (L4440) animals (B) exposed to DMSO]. *p* values for the comparison of the indicated conditions are presented.
(XLSX)

Table S3 **Samples sizes for the assays.**
(XLSX)
